# Acquired Immunodeficiency from Maternal Chemotherapy and Severe Primary *Pneumocystis* Infection in an Infant

**DOI:** 10.1155/2020/5740304

**Published:** 2020-03-15

**Authors:** Adeline Yi Ling Lim, Adrian Christian Mattke, Julia Elizabeth Clark, Alberto Pinzon-Charry, Nelson Alphonso, Nitin Kapur

**Affiliations:** ^1^Department of Respiratory and Sleep Medicine, Queensland Children's Hospital, 501 Stanley Street, South Brisbane, Queensland 4101, Australia; ^2^Department of Pediatric Intensive Care, Queensland Children's Hospital, 501 Stanley Street, South Brisbane, Queensland 4101, Australia; ^3^Pediatric Critical Care Research Group, University of Queensland, St Lucia, Brisbane, Queensland 4072, Australia; ^4^Department of Infectious Diseases, Infection Management Prevention Service, Queensland Children's Hospital, 501 Stanley Street, South Brisbane, Queensland 4101, Australia; ^5^School of Clinical Medicine, Children's Health Queensland Clinical Unit, University of Queensland, St Lucia, Brisbane, Queensland 4072, Australia; ^6^Queensland Pediatric Immunology and Allergy Service, Queensland Children's Hospital, 501 Stanley Street, South Brisbane, Queensland 4101, Australia; ^7^School of Science, Griffith University, Brisbane, QLD 4101, Australia; ^8^Cardiac Surgery, Queensland Pediatric Cardiac Service, Queensland Children's Hospital, 501 Stanley Street, South Brisbane, Queensland 4101, Australia

## Abstract

*Pneumocystis jirovecii* is recognized as an opportunistic pathogen in immunosuppressed patients. We report a case of severe *Pneumocystis* pneumonia (PCP) in an infant with acquired combined immunodeficiency secondary to maternal chemotherapy exposure during the second and third trimesters of pregnancy. The infant required cardiorespiratory support with veno-venous extracorporeal membrane oxygenation (VV-ECMO) for severe respiratory failure. This case highlights the potential for severe acquired immunodeficiency in this patient cohort and further postnatal surveillance is highly recommended.

## 1. Introduction

*Pneumocystis jirovecii* infection is a major cause of morbidity and mortality in children and adults. It is associated with acquired immune deficiency syndrome (AIDS) and recognized as an opportunistic pathogen in other immunosuppressive conditions including allogeneic hematopoietic stem cell transplantation (HSCT), solid organ transplantation, recipients of corticosteroids or immunosuppressive agents, and congenital immunodeficiency syndromes [1–3]. However, recent studies have also indicated that colonisation with *Pneumocystis jirovecii* is common, with reported incidence between 12 and 26% in early infancy [4–6], and immunocompetent infants with *Pneumocystis jirovecii* may present with a mild respiratory infection [7].

Rituximab, a chimeric (mouse/human) monoclonal antibody directed against B-cell surface antigen CD20, is approved for the treatment of B-cell non-Hodgkin's lymphoma (NHL) in combination with CHOP (cyclophosphamide, doxorubicin hydrochloride, vincristine, and prednisolone) therapy or other anthracycline-based chemotherapy regimens [8]. While various complications from rituximab have been published [9], there are few studies on the use of these chemotherapeutic agents during pregnancy and its associated fetal complications. Here, we report an infant with acquired combined immunodeficiency from maternal R-CHOP (rituximab, cyclophosphamide, doxorubicin hydrochloride, vincristine, and prednisolone) therapy during pregnancy, resulting in severe *Pneumocystis* pneumonia (PCP), who required prolonged veno-venous extracorporeal membrane oxygenation (VV-ECMO) to manage the severe respiratory failure.

## 2. Case Report

An 11-week-old female ex-premature infant (corrected gestational age 6 weeks) presented to a peripheral hospital with respiratory distress and a three-day history of cyanotic episodes, following three weeks of upper respiratory tract infection symptoms. The infant was born at 35 weeks' gestation, weighing 2489 g, and did not require resuscitation or respiratory support at birth. The newborn had a 5-day admission to the special care unit for feeding establishment and observation prior to being discharged home well on day 7 of life. The child's mother had received six cycles of R-CHOP for NHL between 17 and 33 weeks of gestation.

The infant was tachypneic and hypoxic, was commenced on high flow nasal cannula support, intravenous (IV) ampicillin and gentamicin, and managed at the peripheral hospital's critical care unit. Over the next two days, respiratory support was escalated to noninvasive ventilation, and antibiotics were changed to IV cefotaxime (D1–D8) and IV azithromycin (D3–D5).

**Figure 1:**
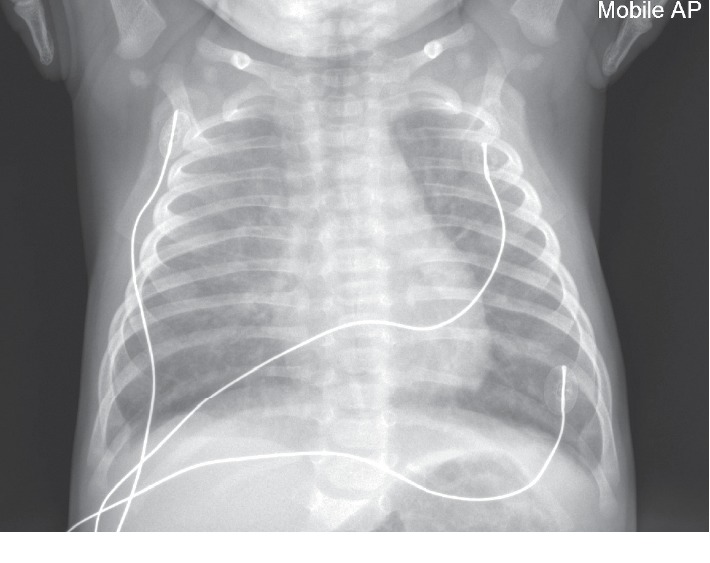
Chest X-ray of infant on presentation with diffuse bilateral ground glass changes.

Nasopharyngeal aspirate (NPA) was negative for eight respiratory viruses. Respiratory virus detection for influenza A, influenza B, parainfluenza 1, 2, and 3, respiratory syncytial virus (RSV), and adenovirus was performed using an in-house respiratory virus multiplex real-time RT-PCR assay [10], while human metapneumovirus (HMPV) was tested with the in-house real-time RT-PCR assay utilising oligonucleotides [11]. Testing for *Pneumocystis jirovecii* was also requested on the NPA specimen and performed using an in-house real-time RT-PCR assay [12]. A synthetic reference standard was also utilised to enable quantification. Human immunodeficiency virus (HIV) serology was negative. A chest X-ray revealed diffuse bilateral ground glass changes (Figure 1).

As the hypoxic respiratory failure worsened, atypical infections and possible childhood interstitial lung disease (ChILD) were considered and pulse IV methylprednisolone was commenced. The NPA was subsequently positive for *Pneumocystis jirovecii* DNA at 2.0 × 10^6^ copies/mL. Oral co-trimoxazole 20 mg/kg/day (D4) was commenced and transitioned to IV co-trimoxazole (D8) due to clinical deterioration.

On day 9, the infant developed a left pneumothorax and pneumomediastinum, requiring intercostal drainage. The child was intubated, ventilated, and retrieved to our Pediatric Intensive Care Unit (PICU) due to worsening respiratory failure. In addition to IV co-trimoxazole, IV lincomycin and primaquine were commenced as second-line PCP therapy due to the clinical deterioration [13], and antimicrobial cover was broadened to IV piperacillin-tazobactam.

Steroids were weaned over the next 10 days (D4–D14). All other microbiological and virological testing were negative. The immune screen revealed mild pan lymphocytopenia, severe hypogammaglobulinemia, and neutropenia, suggestive of a combined immunodeficiency (Table 1). The infant was commenced on fungal (fluconazole) and viral (valaciclovir) prophylaxis as well as immunoglobulin replacement therapy (IRT) and intravenous immunoglobulin (IVIg) at 0.4 g/kg. Granulocyte-colony stimulating factor (G-CSF) was also administered.

The patient's respiratory failure continued to worsen, and the infant subsequently developed acute respiratory distress syndrome (ARDS). An attempt at high-frequency oscillatory ventilation was ineffective. The infant was then placed on VV-ECMO on day 15 of the presentation. Bronchoscopy performed on day 16 was still positive for *Pneumocystis jirovecii* DNA at 1.6 × 10^5^ copies/mL.

From day 40 (24 days after commencement of VV-ECMO) until day 42, the infant received three doses of poractant alfa (Curosurf) via the endotracheal tube. The patient was also commenced on IV hydrocortisone (1 mg/kg 6 hourly) on day 41, which was slowly weaned off over a two-week period. The child was successfully decannulated to conventional ventilation (D46) and extubated on day 53. Subsequent immunological assessment revealed adequate lymphoproliferation responses to phytohemagglutinin (PHA) and adequate levels of recent thymic emigrants. The lymphocyte counts slowly normalised, confirming a secondary combined immune deficiency.

The infant improved clinically, was stepped down to oral antimicrobials, and was discharged home on day 103 with supplemental oxygen. The patient remained on supplementary oxygen at home, IRT, and antimicrobial prophylaxis including oral valaciclovir, fluconazole, and co-trimoxazole until the age of 6 months. The timeline of events is summarised in Table 2. The infant's mother remained well throughout and did not have any investigations for *Pneumocystis jirovecii*. Informed consent was obtained from the infant's parent for this case report.

## 3. Discussion

Most initial exposures to *Pneumocystis jirovecii* occur during the first few months of life, and immunocompetent infants usually have subclinical infections [14] or self-limiting upper respiratory tract infections, although in very young infants, it may present as an invasive pneumonia [15–17].

Immune competent hosts with a combination of innate and adaptive (cellular and humoral) immune responses usually clear *Pneumocystis jirovecii* infections with minimal lung damage [1]. In immune-defective hosts, the *Pneumocystis jirovecii* infection can progress and result in diffuse lung injury secondary to a dysfunctional immune response [1]. The route of *Pneumocystis jirovecii* transmission is unclear where both mothers [6, 18] and healthy infants [7] have been postulated but not proven as reservoirs for transmission. To our knowledge, this is the first reported case of PCP in an infant due to acquired combined immunodeficiency resulting from maternal chemotherapy. ECMO as salvage therapy in immunodeficient children is also uncommon, adding to the uniqueness of this particular case.

Like other monoclonal antibodies, rituximab contains an immunoglobulin G1ĸ (IgG1ĸ) construct and can therefore cross the placenta [19]. When administered during the second trimester of pregnancy, serum rituximab levels have been reported to be similar in both the infant and mother at delivery [20]. Unlike rituximab, transplacental transfer of other chemotherapeutic agents (i.e., doxorubicin, epirubicin, vinblastine, and 4-hydroxy-cyclophosphamide) appears limited, although it has been reported to be variable in animal models [21].

The administration of rituximab usually results in rapid and sustained depletion of the recipient's peripherally circulating CD20+ B cells for approximately 6 months after infusion [8, 20, 22, 23]. In a recent review of the outcomes of neonates with in utero rituximab exposure, a majority of the newborns had B-cell depletion at birth, which normalised by 4–6 months of age [24], and mild lymphopenia has also been reported [25]. Two of these studies also revealed low IgG, IgA, and IgM levels at birth with normalization by 5 weeks to 6 months of age [24]. In keeping with previous reports, our patient's B-cell counts were reduced [20, 26]. Our patient also had significantly decreased T-cell counts, suggestive of an acquired immunodeficiency impairing both cellular and humoral immune pathways.

Our patient had severe hypogammaglobulinemia with unmeasurable IgG levels. Fetal IgG levels usually rise slowly during the second trimester, reach maternal serum concentrations by approximately 26 weeks of gestation, and have maximal transplacental transfer during the last 4 weeks of gestation [19]. Interestingly, in some patients receiving R-CHOP therapy, the maternal total serum immunoglobulin concentrations were not decreased despite an almost complete loss of maternal peripheral B cells [20]. While it is difficult to directly link rituximab (R) or any other chemotherapeutic agents (CHOP) as causative for the presentation in our case, the early postnatal presentation combined with the delayed, yet complete immune reconstitution indicates that the combined immune deficiency was most likely secondary to the maternal chemotherapy.

This infant presented with a severe opportunistic infection in the context of significant lymphocytopenia and severe hypogammaglobulinemia after immune-suppressive maternal chemotherapy. Several hypotheses can be proposed for the infant's presentation including (i) impaired transplacental IgG transfer and/or (ii) direct R-CHOP toxicity in the fetus including transplacental rituximab/CHOP. A significant increased risk of PCP is increasingly recognized in adult lymphoma patients receiving rituximab containing chemotherapy [27]. As presence of B cells was found to be vital in a mouse model for the generation of CD4+ T cells [28], it is possible that a reduction of B cells due to rituximab could lead to insufficient generation of CD4+ T cells and a higher risk for *Pneumocystis jirovecii* infection.

While the adverse effects of rituximab on the recipient have been well described, data on neonatal adverse effects are sparse [15]. Four cases of perinatal infections were previously reported by Chakravarty et al. [9].

In a recent study, the survival rate of pediatric patients with PCP requiring ECMO was higher in the Stockholm programme cohort (89%) versus the Extracorporeal Life Support Organisation (ELSO) Registry cohort (51%) [29]. Despite the limited data available in the pediatric population, this case highlights that survival in an immunocompromised patient with severe lung disease who is supported with VV-ECMO for several weeks is possible and should be considered.

Glucocorticoid treatment for severe ARDS remains controversial [30, 31]. In our case, we elected to use a modified glucocorticoid protocol adapted from the Meduri et al. study [32] to treat the ARDS prior to weaning the patient from extracorporeal support.

## 4. Conclusion

Rituximab is known to cause immunosuppression in the recipient, although its short- and long-term effects on the fetal immune function are not well reported. This case highlights the potential for severe acquired immunodeficiency, leading to severe PCP and subsequent ARDS in an infant from maternal chemotherapy administration in the second and third trimesters of pregnancy. This case also demonstrates that prolonged extracorporeal support can be used successfully in these patients despite severe immunodeficiency and atypical infections. Withdrawal of life-sustaining measures should only be considered after extended periods of time and pharmacological lung maturation attempts.

## Figures and Tables

**Table 1:** Immune screen of the infant early in the patient's presentation.

Investigations	Results	Reference ranges
Neutrophil count (×10^9^/L)	0.29 (low)	1–8.5
Lymphocyte count (×10^9^/L)	1.95 (low)	4–12
CD3 T cells (×10^9^/L)	1.40 (low)	2.3–6.5
CD4+/CD3+ (helper T cells) (×10^9^/L)	1.11 (low)	1.5–5
CD8+CD3+ (cytotoxic T cells) (×10^9^/L)	0.29 (low)	0.5–1.6
CD19 (total B cells) (×10^9^/L)	0.47 (low)	0.6–3
CD56/CD16 (NK cells) (×10^9^/L)	0.05 (low)	0.1–1.3
IgG (g/L)	<1 (low)	2–7.5
IgA (g/L)	<0.07	<0.5
IgM (g/L)	0.3 (low)	0.1–0.7
Phytohemagglutinin test (PHA)	Normal	

**Table 2:** Schema of events from maternal diagnosis of B-cell lymphoma, the birth of the infant, and readmission for *Pneumocystis* pneumonia with the associated management.

Events	Period	Treatment
Pregnancy	(i) 2nd trimester	(i) Diagnosed B-cell lymphoma
	(ii) 17–33 weeks' gestation	(ii) Received 6 cycles of R-CHOP

Birth	(i) At birth (35 weeks' gestation)	(i) Admitted to the special care unit
	(ii) D7	(ii) Discharged home

Readmission	(i) 11 weeks (corrected 6 weeks)	(i) Presented to the peripheral hospital
	(ii) D1	(ii) High flow nasal cannula, IV ampicillin and gentamicin
		(a) Changed to IV cefotaxime (D1–8)
	(iii) D3	(iii) Noninvasive ventilation
		(a) IV azithromycin added (D3–D5)

*Pneumocystis jirovecii* diagnosis and management	(i) D4	(i) *Pneumocystis jirovecii* positive on NPA; PO co-trimoxazole started
	(ii) D8	(ii) Changed to IV co-trimoxazole
	(iii) D9	(iii) Intubated, transferred to tertiary hospital
	(iv) D11	(iv) High-frequency oscillation, commenced 2^nd^ line PCP treatment

Further management	(i) D15	(i) Commenced VV-ECMO
	(ii) D16	(ii) Bronchoscopy—positive for *Pneumocystis jirovecii*
	(iii) D46	(iii) Decannulated to conventional ventilation
	(iv) D53	(iv) Extubated to high flow
	(v) D103	(v) Discharged home on supplemental oxygen
